# Fingerprint Analysis and Comparison of Activity Differences of Crude Venom from Five Species of Vermivorous Cone Snail in the South China Sea

**DOI:** 10.3390/md23030102

**Published:** 2025-02-25

**Authors:** Shibo Sun, Yanling Liao, Jinxing Fu, Yanxia Liang, Yurong Chen, Kailin Mao, Bingmiao Gao

**Affiliations:** Engineering Research Center of Tropical Medicine Innovation and Transformation of Ministry of Education, Hainan Key Laboratory for Research and Development of Tropical Herbs, International Joint Research Center of Human-Machine Intelligent Collaborative for Tumor Precision Diagnosis and Treatment of Hainan Province, School of Pharmacy, Hainan Medical University, Haikou 571199, China; sunshibo@hainmc.edu.cn (S.S.); liaoyanling@hainmc.edu.cn (Y.L.); hy0207145@muhn.edu.cn (J.F.); 15708919030@163.com (Y.L.); hy0215005@muhn.edu.cn (Y.C.)

**Keywords:** cone snail, conotoxins, insecticidal activity, analgesic activities, anti-proliferative

## Abstract

The South China Sea is rich in cone snail resources, known for producing conotoxins with diverse biological activities such as analgesic, anticancer, and insecticidal effects. In this study, five vermivorous cone snail samples were collected from the South China Sea and their crude venom was extracted to investigate the variations in venom components and activities, aiming to identify highly active samples for further research. Cluster analysis using reverse-phase high-performance liquid chromatography (RP-HPLC) fingerprints and mitochondrial cytochrome c oxidase I (COI) gene sequences revealed that the diversity of venom components across different conotoxin species is genetically correlated. Activity assays demonstrated that all five cone snail venoms exhibited lethal effects on insects and zebrafish. Notably, the crude venom of *Conus quercinus* showed the highest insecticidal activity with an LD_50_ of 0.6 μg/mg, while *C. tessellatus* venom exhibited the most potent zebrafish lethality with an LD_50_ of 0.2 μg/mg. Furthermore, the crude venom from four cone snail species demonstrated toxicity against ovarian cancer cells, and only *C. caracteristicu* venom displayed significant analgesic activity. This study systematically identifies cone snail samples with promising insecticidal, anticancer, and analgesic properties, paving the way for the development and utilization of cone snail resources from the South China Sea and offering a novel approach for advancing marine peptide drug research.

## 1. Introduction

Cone snails, marine gastropods belonging to the family Conidae, are distributed across the world’s tropical coastal waters, with a significant presence in the South China Sea. These marine gastropods exhibit a remarkable diversity in their distribution, with some species found across the entire tropical Indo-Pacific, while others are restricted to specific bays or seamounts [1,2,3]. It is estimated that there are around 900 species within the Conus genus, making it the largest genus among marine invertebrates [4]. The greatest species richness for cone snails is observed in the Philippines and countries towards Papua New Guinea, the Solomon Islands, New Caledonia, and Fiji [5]. However, the South China Sea also harbors a significant number of these species, highlighting its importance as a biodiversity hotspot for cone snails. Cone snails are renowned for their complex venom delivery systems and the array of potent toxins they produce [6]. These fascinating creatures can be categorized into three distinct trophic types based on their predation habits: vermivorous (worm-eating), piscivorous (fish-eating), and molluscivorous (mollusk-eating) [7,8]. Among the vast diversity of cone snails, the South China Sea boasts an abundant resource of genus Conus, with the vermivorous cone snails being particularly prevalent. The *Conus betulinus*, or “bucket snails”, are the most numerous in this region. Recent advancements in genomics, transcriptomics, and peptidomics have shed light on the intricate composition of these venoms, revealing over 133 distinct cone snail toxins [7,9,10,11]. However, predicting their pharmacological activities remains a challenge due to the vast diversity and complexity of these bioactive peptides.

Cone snail toxins, also known as conotoxins or conopeptides, are a diverse group of bioactive peptides produced by predatory marine cone snails [12,13,14]. These toxins are characterized by their potency, specificity, and unique pharmacological properties [15,16,17]. Conotoxins are rich in disulfide bridges, which contribute to their stability and diversity. They are known for their extraordinary potency and high specificity, with the ability to target specific ion channels, receptors, and transporters with remarkable precision [15,18,19]. Conotoxins have been classified into pharmacological families based on their targets, such as α, δ, μ, ω, κ, γ, etc., [18,20,21,22,23,24,25]. For instance, ω-conotoxins are antagonists of voltage-gated calcium channels, and some are effective against neuropathic pain [21]. The ω-conotoxin MVIIA, derived from the fish-hunting species *C. magus*, is a synthetic version known as Ziconotide and is used to treat severe pain [26]. Different cone snail species exhibit a remarkable diversity in their venom composition. Each of the approximately 900 cone snail species expresses a unique library of hundreds of peptide toxins. This diversity is a result of evolutionary adaptation, with each species developing its own set of conopeptides to target the specific physiological circuits of their prey [4]. It is estimated that there may be over 50,000 different conopeptides across all cone snail species [27], highlighting the vast potential for novel drug discovery and therapeutic applications.

In this study, we aim to address the central question of how the venom composition and biological activities of five species of vermivorous cone snail from the South China Sea differ, with a particular focus on their insecticidal, anticancer, and analgesic properties. This research is crucial as it explores the potential of cone snail venoms as a source of novel marine drugs, which could lead to significant advancements in pharmaceutical applications. To achieve this, we utilized reverse phase high-performance liquid chromatography (RP-HPLC) for venom fingerprinting and mitochondrial cytochrome c oxidase I (COI) gene clustering to analyze the genetic diversity of the cone snails, followed by conducting a series of bioactivity assays. This comprehensive approach not only enhances our understanding of vermivorous cone snail venom diversity but also lays the groundwork for future drug development initiatives.

## 2. Results

### 2.1. Protein Components in the Crude Venoms

**Figure 1:**
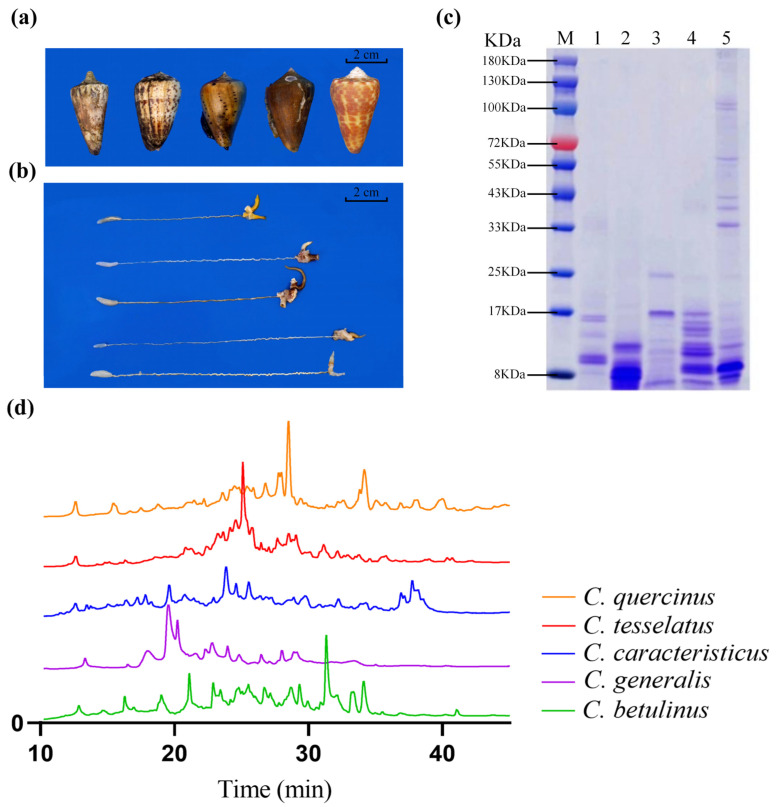
Comparison of external characteristics and crude venom composition of five species of vermivorous cone snails. (**a**) External characteristics of common vermivorous cone snails found in the South China Sea. From left to right: *C. generalis*, *C. caracteristicus*, *C. betulinus*, *C. quercinus*, and *C. tesselatus*. (**b**) Schematic representation of venom tube dissection for the five species of cone snail. From top to bottom: venom glands and ducts of *C. generalis*, *C. caracteristicus*, *C. betulinus*, *C. quercinus*, and *C. tesselatus*. (**c**) SDS-PAGE analysis of crude venom from the five species of cone snail. M indicates the standard marker. Lanes 1–5 correspond to the crude venom of *C. tesselatus*, *C. caracteristicus*, *C. quercinus*, *C. betulinus*, and *C. generalis*, respectively, with a loading volume of 10 μL. (**d**) RP-HPLC analysis of crude venom from the five species of cone snail.

The South China Sea is rich in cone snail biodiversity. In the Sanya region, we collected specimens and identified five predominant vermivorous cone snail species: *C. generalis*, *C. caracteristicus*, *C. betulinus*, *C. quercinus*, and *C. tesselatus* (Figure 1a) The schematic diagram of dissecting the venom tube shows that the radula morphology of the five species of vermivorous cone snails is different, and there are significant differences in the size and length of the venom tube and muscle vesicles among different cone snails (Figure 1b). Specifically, the venom tube of the *C. generalis* is the most compact, measuring a mere 8.3 cm in length, while the *C. betulinus* possesses the most elongated venom tube among the species studied, stretching to an impressive 15.6 cm. The protein content of the crude venom was separated using Sodium Dodecyl Sulfate–PolyAcrylamide Gel Electrophoresis (SDS–PAGE); as shown in Figure 1c, the protein molecular bands are primarily concentrated below 17 kDa. Notably, the venom of *C. tesselatus* exhibits a broader distribution of molecular bands, with some bands extending up to 100 kDa. The complexity of crude venom composition is further underscored by the results of RP-HPLC, as shown in Figure 1d. The primary peaks, indicative of venom composition, are concentrated between 15 and 40 min, highlighting the complex characteristics of these venom compounds.

### 2.2. Similarity Evaluation and Phylogenetic Tree Analysis

Ten batches of crude venom solutions across five species of vermivorous cone snails were taken for analysis. Using the sample chromatogram as the reference fingerprint, multi-point calibration was performed to generate overlay and control fingerprints (see Figures S1–S5). Analysis revealed 16 common peaks in the crude venom of *C. generalis* and *C. caracteristicus*, 18 in *C. betulinus*, 14 in *C. tesselatus*, and 15 in *C. quercinus*. The similarity evaluation results can be found in Tables S1–S5, where all the similarity values of samples from different species are larger than 0.917. This indicates that the quality of five species of vermivorous cone snail samples from the South China Sea is relatively stable, with similar chemical composition but varying content.

**Figure 2:**
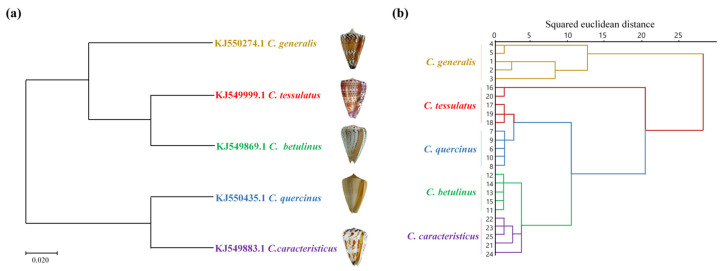
Cluster analysis of mitochondrial COI gene sequence and HPLC fingerprint of five species of vermivorous cone snails. (**a**) Based on COI gene sequences of five species of vermivorous cone snails, a phylogenetic tree was constructed using the ML method. (**b**) Cluster diagram based on RP-HPLC data of five species of crude venom from vermivorous cone snails.

Given the genetic disparities in the chemical composition of cone snail venom, a phylogenetic tree was constructed using mitochondrial COI gene sequences. This analysis aimed to correlate venom compositional variations with genetic divergence. The morphologically defined species were segregated into discrete clusters within the phylogenetic tree, as inferred from the cone snail COI gene fragment. As shown in Figure 2a, the phylogenetic analysis revealed that among the five species of vermivorous cone snail, *C. caracteristicus* and *C. quercinus* exhibited the closest genetic relationship, sharing a common branch in the Maximum Likelihood (ML) tree derived from COI sequences. *C. generalis* emerged as the earliest diverging lineage among the other three species, with *C. tesselatus* and *C. betulinus* displaying a strong genetic similarity. Additionally, the crude venom RP-HPLC cluster analysis depicted in Figure 2b indicated that at a squared Euclidean distance threshold of 25, the species formed distinct clusters. Notably, *C. betulinus* and *C. caracteristicus* were the first to cluster, followed by the grouping of *C. tesselatus* and *C. quercinus*, which eventually merged with the cluster that included *C. generalis*.

The cluster analysis of the COI gene and RP-HPLC fingerprinting largely align, except for a distinction where *C. betulinus* and *C. caracteristicus* are grouped differently. This suggests a correlation between venom patterns and genetic diversity. Furthermore, these findings offer evidence for a reliable experimental approach to developing chemical fingerprint spectral clustering trees.

### 2.3. Insect Toxicity

**Figure 3:**
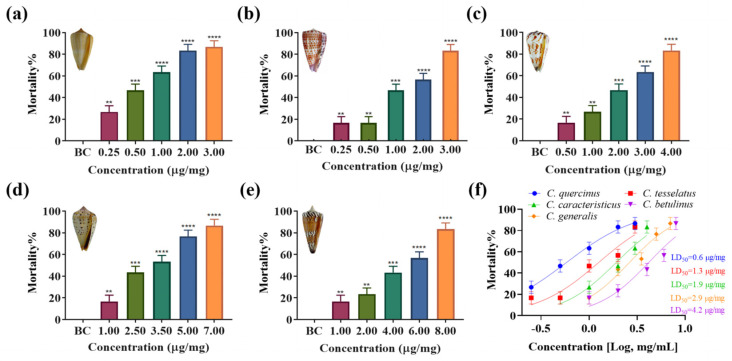
Insecticidal effects and LD_50_ values of crude venom from five species of vermivorous cone snail on *Spodoptera litura* larvae at various concentrations. (**a**–**e**) The insecticidal effects of crude venoms from *C. quercinus*, *C. tesselatus*, *C. caracteristicus*, *C. betulinus*, and *C. generalis* on *Spodoptera litura* at a specified concentration, *n* = 3. (**f**) The LD_50_ values of these five species of vermivorous cone snail against *Spodoptera litura* larvae at varying concentrations. ** *p* < 0.01, *** *p* < 0.001, **** *p* < 0.0001 versus blank control (BC) group.

To evaluate the lethal effects of venom from five species of vermivrous cone snails (*C. generalis*, *C. caracteristicus*, *C. betulinus*, *C. quercinus*, and *C. tesselatus*) on *Spodoptera litura* larvae in vivo, crude venom extracts were administered into the lower abdomen of the larvae. As illustrated in Figure 3, crude venom significantly increased the mortality rate of *Spodoptera litura* larvae in a concentration-dependent manner. Specifically, *C. quercinus* and *C. tesselatus* exhibited significant insecticidal activity even at low venom concentrations (at a concentration of 1.00 μg/mg, *C. quercinus* and *C. tesselatus* venom resulted in a mortality rate of 64% ± 10% and 48% ± 10%, respectively), with a marked increase in mortality rates as the venom dosage increased. In contrast, the venom of *C. generalis* and *C. betulinus* showed lower insect toxicity (at a concentration of 1.00 μg/mg, *C. generalis* and *C. betulinus* venom resulted in a mortality rate of 18% ± 10% and 18% ± 10%, respectively), requiring higher concentrations to achieve statistically significant lethal effects compared to the other three species. The median lethal dose (LD_50_) values are presented in Figure 3f. Notably, *C. quercinus* exhibits the highest toxicity, with an LD_50_ value of 0.6 μg/mg, while *C. generalis* shows the lowest toxicity, with an LD_50_ value of 4.2 μg/mg.

### 2.4. Cytotoxic Activity

**Figure 4:**
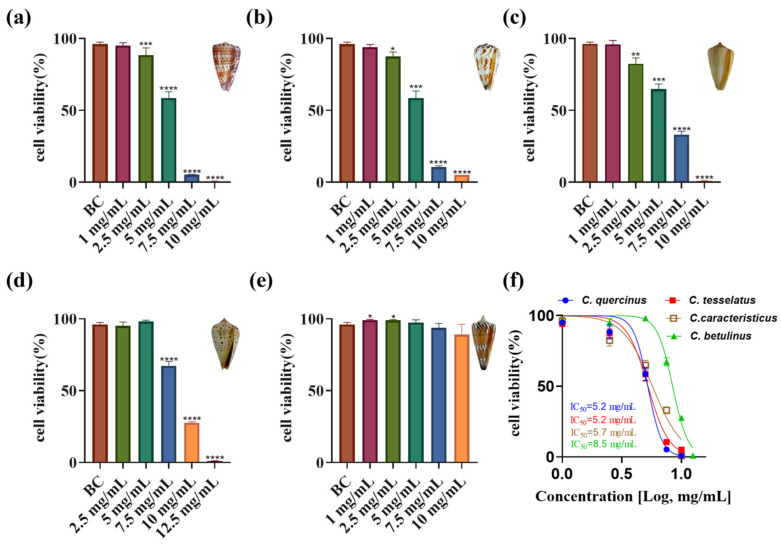
Cytotoxicity assays of crude venom from five species of vermivorous cone snail on ovarian cancer cells at different concentrations. (**a**–**e**) Cytotoxicity assays of crude venom from *C. quercinus*, *C. tesselatus*, *C. caracteristicus*, *C. betulinus*, and *C. generalis* on ovarian cancer cells at predetermined concentrations, *n* = 3. (**f**) IC_50_ value for the crude venom of four species of vermivorous cone snail on ovarian cancer cells at varying concentrations. * *p* < 0.05, ** *p* < 0.01, *** *p* < 0.001, **** *p* < 0.0001 compared to the blank control (BC) group.

The impact of crude venom from various cone snail species on ovarian cancer cell viability was assessed, revealing significant differences in anti-proliferative activity (Figure 4a–e). Increasing doses of crude venom from *C. quercinus, C. tessellatus, C. caracteristicus,* and *C. betulinus* corresponded with lower viability rates in ovarian cancer cells. Notably, the venom of *C. tessellatus and C. quercinus* exhibited the strongest toxicity, with an IC_50_ value of 5.2 mg/mL, indicating significant anti-proliferative activity (*p* < 0.05). In contrast, the crude venom of *C. generalis* showed an insignificant impact on ovarian cancer cell inhibition, with no statistically significant difference in activity compared to the control (*p* = 0.1673), and thus its IC_50_ value was not determined in this study. The IC_50_ values for the other species were as follows: *C. caracteristicus* at 5.7 mg/mL, and *C. betulinus* at 8.5 mg/mL (Figure 4f).

### 2.5. Toxicity Assessment on Adult Zebrafish

**Figure 5:**
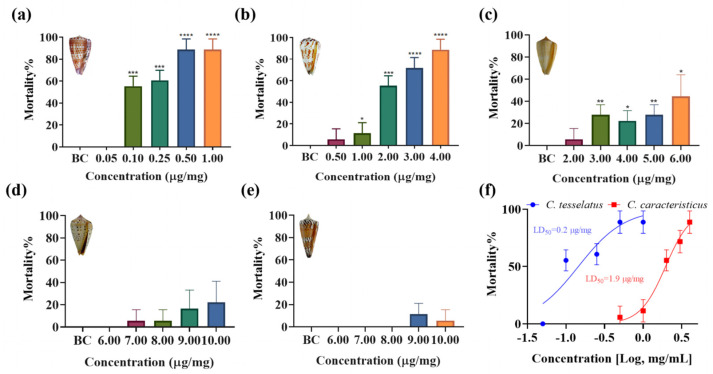
Toxicity analysis of crude venom from five species of vermivorous cone snail on zebrafish at varying concentrations. (**a**–**e**) The mortality rates of zebrafish exposed to crude venom from *C. tesselatus*, *C. caracteristicus*, *C. quercinus*, *C. betulinus*, and *C. generalis* at specific concentrations, *n* = 3. (**f**) The LD_50_ values for the crude venom of *C. tesselatus* and *C. caracteristicus.* * *p* < 0.05, ** *p* < 0.01, *** *p* < 0.001, **** *p* < 0.0001 versus the blank control (BC) group.

To assess the toxicity of crude venom from five species of vermivorous cone snail on vertebrate models, experiments were conducted using adult zebrafish. Figure 5 highlights significant differences in zebrafish mortality rates when exposed to crude venoms from five species of cone snails, revealing a measurable impact on survival rates compared to a blank control group (*p* < 0.05). Among the venoms tested, *C. tesselatus* exhibited the highest lethal activity, inducing higher mortality rates at lower concentrations (at a concentration of 1.00 μg/mg, *C. tesselatus* venom resulted in a mortality rate of 86% ± 10%), higher than *C. caracteristicus* (at a concentration of 1.00 μg/mg, *C. caracteristicus* venom resulted in a mortality rate of 12% ± 10%), with rapid onset of motility disorders. The LD_50_ value for *C. tesselatus* was determined to be 0.2 μg/mg, indicating its high potency, whereas *C. caracteristicus* had a higher LD_50_ value of 1.9 μg/mg, suggesting lower potency. Mortality rates increased significantly with higher venom concentrations, a trend more pronounced for *C. tesselatus* compared to *C. caracteristicus*, reinforcing the higher lethality of *C. tesselatus* venom. In contrast, the venoms of *C. quercinus*, *C. betulinus*, and *C. generalis* exhibited minimal or negligible toxicity, with low or absent mortality rates across tested concentrations, making it impossible to determine IC_50_ values due to insufficient mortality.

### 2.6. Assessment of Nociceptive-like Swimming Behavior in Larval Zebrafish

Building on the understanding of the analgesic effects of crude venoms from five species of vermivorous cone snails, behavioral assays were conducted on zebrafish larvae to evaluate these effects. Zebrafish larvae were selected due to their observable locomotor behaviors, which can be effectively tracked. The larvae were exposed to crude venoms from *C. tesselatus*, *C. caracteristicus, C. quercinus*, *C. betulinus*, and *C. generalis*, each at a concentration of 5, 10, and 20 μg/mL, respectively, and their locomotor activity was analyzed.

**Figure 6:**
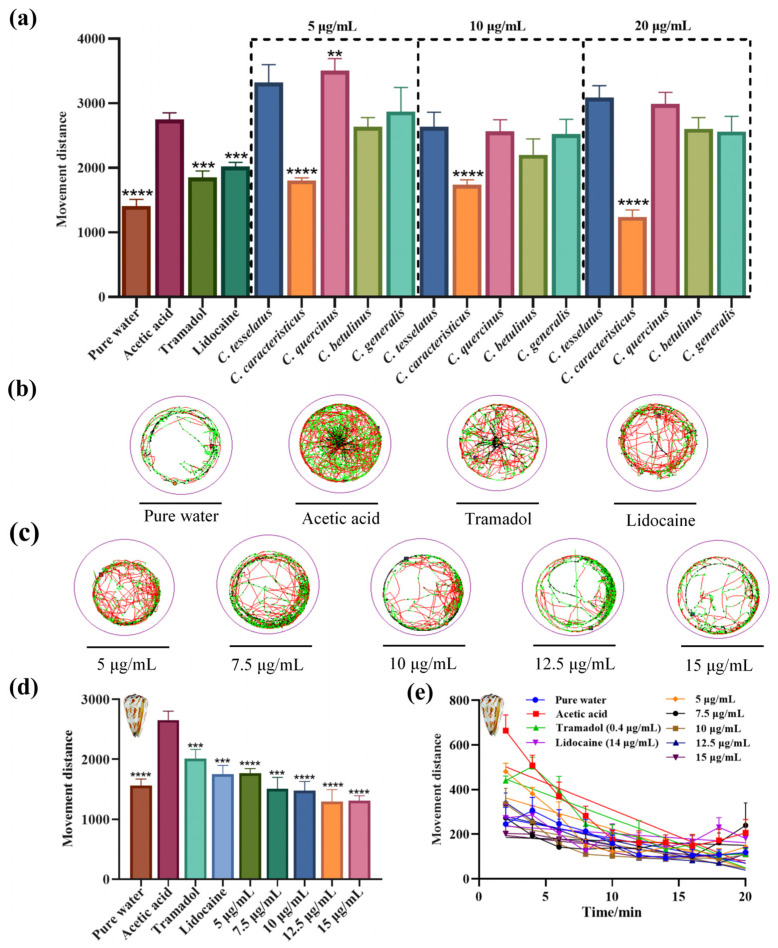
Analgesic effects of crude venoms from five species of vermivorous cone snail on zebrafish larvae. (**a**) Analgesic effects of crude venoms from five species of vermivorous cone snail on zebrafish larvae, evaluated based on their swimming distance, *n* = 12. (**b**) Motion trajectory diagrams for the blank control group, acetic acid model group, and positive drug (Tramadol and Lidocaine) group. Motion trajectories were recorded every 5 s, with instantaneous velocity indicated by color: black (<2 mm/s), green (2–8 mm/s), and red (>8 mm/s). (**c**) Motion trajectory diagram of zebrafish larvae treated with crude venom from *C. caracteristicus*. (**d**) Analgesic effects of *C. caracteristicus* crude venom on zebrafish larvae, *n* = 12. (**e**) Line graph showing movement changes in zebrafish larvae treated with *C. caracteristicus* venom, measured every two minutes. ** *p* < 0.01, *** *p* < 0.001, **** *p* < 0.0001 versus the acetic acid group.

Increased locomotor activity in zebrafish larvae in response to acetic acid may be interpreted as a nocifensive-like escape behavior in response to a noxious stimulus. Lidocaine, a widely recognized non-selective sodium channel blocker, offers rapid pain relief, making it ideal for evaluating immediate analgesic effects., while tramadol, with its dual mechanism as a μ-opioid receptor agonist, provides potent central analgesia [28]. The acetic acid group elicited an acute and rapid swimming behavior immediately upon treatment (exhibited a mean movement of 2750 ± 150 mm over 20 min), while the tramadol (moved a mean distance of 1850 ± 140 mm) and lidocaine groups (moved a mean distance of 2020 ± 100 mm) showed reduced movement, confirming the model’s validity. Notably, the *C. caracteristicus* venom-treated group demonstrated a significant reduction in movement distance (mean movement distances of 1240 ± 130 mm), similar to the pure water groups (mean movement distances of 1390 ± 140 mm), suggesting its analgesic efficacy (Figure 6a; One-way ANOVA, *p* < 0.0001).

The motion trajectory diagrams (Figure 6b,c) further illustrate these findings, with *C. caracteristicus* venom showing a distinct pattern of reduced locomotion in a dose-dependent manner. The line graph (Figure 6e) highlights the time-dependent changes in movement, reinforcing the analgesic potential of *C. caracteristicus* venom. Statistical analysis confirmed the significance of these observations, with *p*-values indicating strong analgesic effects compared to the acetic acid group.

## 3. Discussion

The genus Conus, with over 900 known species, exhibits remarkable diversity in its venom composition [4]. Each species has evolved to express a unique cocktail of conopeptides, adapted to the biological targets of their specific prey [29,30]. The venom composition varies not only between species but also within individuals of the same species, due to factors such as predatory or defensive needs [16,31,32]. This intraspecific and interspecific venom variation provides a rich source of bioactive peptides but also presents challenges for venom characterization. The pharmacological activity of conotoxins has been extensively studied, with applications ranging from pain management to cancer treatment [33,34,35,36]. Our study aimed to address the question of which pharmacological direction to pursue by examining the venom diversity and identifying potential therapeutic targets.

The COI gene has been widely used in molecular phylogenetic studies of cone snails and other gastropods [3,37,38]. It has helped elucidate evolutionary relationships within the diverse Conus genus [39]. COI, along with other mitochondrial genes including 16S and 12S rRNA, has revealed major clades and subclades within Conus, providing insights into their diet evolution, biogeography, and toxin diversity [40]. Beyond phylogenetics, targeted sequencing of venom genes has shed light on conotoxin evolution, revealing patterns of positive selection, gene expression regulation, and extensive gene turnover across species [41]. In this study, we employed hierarchical cluster analysis to elucidate the relationships among conotoxins based on RP-HPLC fingerprinting data and mitochondrial COI gene sequence homology. Our results demonstrate that the compositional diversity of crude venom is underpinned by genetic variability, with distinct correlations observed among different conotoxin types. These findings significantly contribute to the identification and characterization of novel and potentially more efficacious conotoxins, thereby advancing our understanding of their evolutionary and functional diversity.

Traditionally, vermivorous cone snails are known for their insecticidal activity, with specific conotoxins targeting insect nicotinic acetylcholine receptors (nAChRs) [7,8,9,42]. Our group has explored the potential of conotoxins from vermivorous cone snails as novel insecticides. We have identified and synthesized various conotoxins from species like *C. quercinus* and *C. betulinus*, demonstrating their insecticidal activities through in vitro and in vivo assays [7,8,9]. Both α-conotoxins with two disulfide bonds and cysteine-free conopeptides have shown promising results, with some peptides exhibiting high insecticidal efficacy. Specifically, two cysteine-free peptides from *C. betulinus*, Bt010 and Bt016, showed high efficacy with LD_50_ values of 9.07 nM and 10.93 nM, respectively [8,9]. Molecular docking analyses showed that these conotoxins bind to the central pore of nAChRs, forming multiple hydrogen bonds with key residues like Tyr-112 and Lys-116 [8,9]. Our findings on the vermivorous cone snails corroborate the previous literature [7,8,9], demonstrating their potential as natural insecticides. However, a significant discovery in our study was the substantial toxicity of the crude venom from these cone snails towards zebrafish, as evidenced by the LD_50_ values: *C. tesselatus* venom at 0.2 μg/mg and *C. caracteristicus* venom at 1.9 μg/mg. This toxicity profile contrasts with some previous studies [7,8,9], suggesting a divergence that may be attributed to the complexity and diversity inherent in the crude venom of cone snails. It is plausible that individual insecticidal peptides within the venom possess high selectivity and specificity, which could account for the observed differences in toxicity. Further research is needed to isolate and characterize specific peptides that possess selective insecticidal activity without harming vertebrates.

Cone snail venoms have shown promising anticancer potential across various studies. Extracts from *C. virgo*, *C. vexillum*, *C. textile*, *C. flavidus*, and *C. geographus* demonstrated cytotoxic effects against multiple cancer cell lines, including breast, liver, lung, and ovarian cancers [33,43,44,45,46,47]. Specifically, *C. virgo* venom extracts showed IC_50_ values between 173.89 and 481.27 μg/mL against breast, liver, and colorectal cancer cells [48]. *C. flavidus* venom exhibited potent effects against liver (HepG_2_, IC_50_ = 1.593 ± 0.05 μg/mL) and lung (A_549_, IC_50_ = 7.836 ± 0.4 μg/mL) cancer cells [46]. *C. vexillum* venom from Marsa Alam was effective against lung cancer cells (A_549_, IC_50_ = 4.511 ± 0.03 μg/mL) [46]. Notably, *C. geographus* extract demonstrated broad antiproliferative activity against ovarian (SKOV-3, IC_50_ = 22.7 ± 2.2 μg/mL), breast (MDA-MB-231, IC_50_ = 68.7 ± 6.2 μg/mL; MCF-7, IC_50_ = 47 ± 4.2 μg/mL), and liver (HepG_2_, IC_50_ = 19 ± 2.1 μg/mL) cancer cell lines [47]. Individual conotoxins from various species showed anti-ovarian cancer effects, with IC_50_ values ranging from 24.29 to 111.6 μM [33]. Our study finds that crude venom from different cone snail species exhibits varying levels of anti-proliferative activity against ovarian cancer cells. Among the tested species, *C. tessellatus* showed the strongest toxicity, with an IC_50_ value of 5.238 mg/mL (*p* < 0.05). In contrast, *C. generalis* had no significant effect on ovarian cancer cell viability (*p* = 0.1673). The IC_50_ values for *C. quercinus*, *C. caracteristicus*, and *C. betulinus* were 5.226 mg/mL, 5.711 mg/mL, and 8.496 mg/mL, respectively, highlighting species-specific differences in venom potency.

Peptides with potent analgesic properties found in cone snail venoms present promising opportunities for advancing pain management therapies. Studies have identified novel conotoxins from various Conus species, including *C. coronatus*, *C. frigidus* [49], *C. textile* [50], *C. moncuri* [51], *C. lividus* [49], and *C. marmoreus* [52]. These peptides exhibit analgesic effects in animal models of acute and chronic pain, often comparable to morphine [50]. Mechanisms of action include inhibition of voltage-gated calcium channels [51] and interaction with cannabinoid receptors [53]. The ω-conotoxin MVIIA (Prialt^®^) has been clinically evaluated for severe chronic pain [54]. Combining different conotoxins, such as conantokin-G and ω-conotoxin MVIIA, can produce synergistic antinociceptive effects with reduced side effects [55]. Our study explored the pain-relieving effects of venoms from five cone snail species using zebrafish larvae. The venoms reduced pain-related movement caused by acetic acid in a dose-dependent manner. Among the venoms tested, *C. caracteristicus* showed the strongest analgesic effect, significantly reducing acetic acid-induced hyperlocomotion to levels similar to the Lidocaine group. Statistical analysis validated these results, underscoring the venom’s significant potential as a subject for further investigation.

In conclusion, this study provides a comprehensive investigation into the crude venom extracted from five species of vermivorous cone snails native to the South China Sea, uncovering their diverse biological activities and potential for therapeutic applications. Through detailed analyses of venom composition, phylogenetic relationships, and pharmacological properties, we identified significant insecticidal, anticancer, and analgesic activities across different species. Notably, *C. quercinus* demonstrated the strongest insecticidal activity, *C. tessellatus* exhibited potent anticancer effects, and *C. caracteristicus* showed promising analgesic properties, highlighting the species-specific diversity of these bioactive peptides. The integration of RP-HPLC fingerprinting and COI gene sequence analysis revealed a clear correlation between venom composition and genetic variability, offering valuable insights for future studies on the evolution and functional diversity of conotoxins. These findings not only expand our understanding of the pharmacological potential of cone snail venoms but also underscore the untapped value of vermivorous species in the development of innovative marine-derived drugs. Moving forward, the isolation and characterization of individual peptides from these venoms will be essential for advancing their application in therapeutic and agricultural domains, while also promoting sustainable utilization and conservation of marine biodiversity.

## 4. Materials and Methods

### 4.1. Venom Extraction

Five species of vermivorous cone snails were collected in the Sanya area of the South China Sea (Figure 1a). Crude venom was extracted by cutting and crushing the shells to extract the snail flesh, followed by removing the venom duct (as illustrated in Figure 1b). The venom duct tissue was then cut into small sections (1–2 mm) and stored at a low temperature for further processing. The venom duct tissue was immersed in a 30% acetonitrile aqueous solution and subjected to low-temperature ultrasonication for 5 s. The solution was vortexed thoroughly and the process was repeated five times. The mixture was then centrifuged at 10,000 rpm and 4 °C for 5 min using a high-speed centrifuge, and the supernatant was collected. The remaining venom duct tissue was re-extracted using the same 30% acetonitrile solution following the same procedure [56]. The supernatants from the two extraction cycles were combined and frozen at −80 °C. Finally, the crude venom solution was concentrated using a freeze-dryer, yielding the crude venom from the vermivorous cone snails.

### 4.2. Bicinchoninic Acid (BCA) Protein Assay

Bicinchoninic Acid (BCA) Protein Assay was employed to quantify the protein concentration in the extracted crude venom of vermivorous Conus species [57]. Absorbance values of all samples were measured at a wavelength of 562 nm using an enzyme-linked immunosorbent assay (ELISA) reader, and the readings were recorded. A sample without BSA was used as the blank control. A standard curve was generated with absorbance at 562 nm (A_562_) plotted on the vertical axis and BSA content on the horizontal axis. This standard curve was then used to calculate the protein concentration in each crude venom sample.

### 4.3. SDS-PAGE

SDS-PAGE was performed essentially as described by Laemmli [58]. A total of 10 mg of each crude venom of vermivorous cone snail was weighed and dissolved in 1 mL of deionized water and then centrifuged at 1000 rpm for 5 min. Then, 16 μL of the supernatant was mixed with 4 μL of the sample loading buffer (0.01 M Tris-HCl, 10% (*w*/*v*) SDS, 10% (*v*/*v*) glycerol, 0.1% (*w*/*v*) bromophenol blue, 2% β-mercaptoethanol) and then boiled for 10 min. Samples were loaded onto gel slab consisting of 12% separating gel (pH 8.8) and 4% stacking gel (pH 6.8), and subjected to electrophoresis at 100 V for 1 h. The electrophoresis gels were stained using Coomassie Brilliant Blue R-250.

### 4.4. Chromatographic Conditions

The crude venom fractions of vermivorous cone snails were preliminarily analyzed using RP-HPLC with a UV detector and a C_18_ column (Diamonsil, 250 × 4.6 mm, 5 μm). For each sample, 10 mg of crude venom was dissolved in 1 mL of deionized water and centrifuged at 4 °C and 10,000 rpm for 10 min. The supernatant was filtered through a 0.45 μm microporous membrane, and 100 μL of the filtered sample was injected into the RP-HPLC system using an auto-sampler. The mobile phase consisted of solution A (0.1% trifluoroacetic acid [TFA] in acetonitrile) and solution B (0.1% TFA in ultrapure water). Both solvents were filtered through 0.45 μm filter papers and degassed for 15 min using an ultrasonicator before use. The separation was performed at a constant flow rate of 1 mL/min over 65 min using a linear gradient from 5% to 70% solution A. The elution was monitored by measuring absorbance at 214 nm.

### 4.5. Establishment of Fingerprint and Similarity Analysis

Fingerprint mapping was employed to analyze venom composition differences within and between five species of cone snail (*C. generalis*, *C. betulinus*, *C. caracteristicus*, *C. tesselatus*, and *C. quercinus*) [59]. Crude venom samples were prepared, filtered, and analyzed using RP-HPLC to generate chromatographic fingerprints, with Chromatogram 5 selected as the reference spectrum. The “Similarity Evaluation System for Chromatographic Fingerprint of Traditional Chinese Medicine developed by Chinese Pharmacopoeia Committee (Version 2004A) (Beijing, China)” software was used to calculate fingerprint similarities across 10 batches of venom per species. Cluster analysis, performed in SPSS 26.0 using the relative peak areas of characteristic peaks from 25 samples, produced a dendrogram to assess venom quality, variability, and interspecies relationships.

### 4.6. Phylogenetic Tree Analysis

COI gene sequences of five species of vermivorous cone snails were obtained from the National Center for Biotechnology Information (NCBI) database (http://www.ncbi.nlm.nih.gov/, accessed on 20 November 2024). Representative gene sequences were selected, processed, and analyzed using MEGA-X (v10.2.6) software. The Maximum Likelihood (ML) method was employed to construct an evolutionary phylogenetic tree, illustrating the genetic relationships among the species [60].

### 4.7. Insect Toxicity Test

*Spodoptera litura* eggs were purchased from Guangzhou Zhixin Biotechnology Co., Ltd (GuangZhou, China) and cultured at room temperature to 4–6 days post-fertilization (dpf) for experimental use. The insect toxicity test was conducted using the fourth to sixth instar larvae of *Spodoptera litura*, weighing approximately 180 mg. Prior to the experiment, the larvae were fed with standard feed ad libitum. Five venoms were dissolved in 0.7% saline to the required concentrations. A liquid micro-injector (Shanghai High Pigeon, Shanghai, China) was used to administer 3 μL of the solution into the abdomen of each larva. The control group received an injection of 0.7% saline. Each concentration was tested in triplicate (*n* = 3), with lethality rates determined using 10 larvae per replicate. Mortality of *Spodoptera litura* larvae was observed and recorded over 36 h following the injection of the venom solutions or saline control [7].

### 4.8. Cytotoxicity Assays

The cytotoxic effects of five vermivorous venom on selected human ovarian cancer cells were evaluated using the Cell Counting Kit-8 (CCK-8) assay [61]. Briefly, ovarian cancer cells were harvested and resuspended to prepare a cell suspension. The cells were seeded into 96-well plates at a density of 5×10^4^ cells per well and incubated in a biochemical incubator at 37 °C for 24 h to allow attachment and growth. The five venom samples were dissolved in the culture medium to the desired concentrations and filtered to ensure sterility. Each sample (100 μL) was added to the wells, with three replicates for each concentration. Wells containing only culture medium served as the blank control group. Following a 24 h incubation at 37 °C, 90 μL of culture medium and 10 μL of CCK-8 reagent were added to each well. The plates were then incubated for an additional 10 h. Finally, the optical density (OD) was measured at 450 nm using a Synergy HTX multifunctional mioplate reader (manufactured by Agilent BioTek, Burlington, VT, USA) to assess cell viability.

### 4.9. Adult Zebrafish Toxicity Assay

Three-month-old zebrafish with a standard body length of 2–2.5 cm were used for the toxicity study. Five distinct conotoxin solutions were prepared by dissolving the toxins in 0.7% physiological saline to achieve the desired concentrations. Using a precision microsyringe, 4 μL of each venom solution was injected into the lower abdominal cavity of the zebrafish. To ensure complete delivery of the conotoxin and minimize residual toxin in the syringe, the syringe was rinsed with 10 μL of 0.7% physiological saline, which was subsequently injected into the zebrafish.

Physiological saline was used as the control. Each concentration was tested in triplicate (*n* = 3), with lethality rates determined using six zebrafish per replicate. The zebrafish were maintained in a stable aquatic environment throughout the experiment, and their physiological status was carefully monitored and recorded over a 24 h observation period [62,63].

### 4.10. Locomotor Behavior of Zebrafish Larval

AB series wild-type zebrafish eggs were sourced from the Institute of Hydrobiology, Chinese Academy of Sciences, and incubated in our laboratory’s zebrafish breeding system. The embryos were maintained at 28 °C in an incubator until they reached 3–5 dpf to facilitate behavioral verification. Each juvenile fish was individually placed into a well of a 24-well plate, with ten replicates per treatment group (*n* = 10). Experimental groups were designed as follows: (1) a control group exposed to pure water, (2) a model group treated with pure water and a 0.01% acetic acid aqueous solution, (3) treatment groups exposed to different concentrations of conotoxin crude toxin aqueous solutions (5, 10, and 20 μg/mL) mixed with 0.01% acetic acid aqueous solution, (4) positive control group 1 treated with a tramadol solution (0.4 μg/mL) mixed with 0.01% acetic acid aqueous solution, and (5) positive control group 2 treated with a lidocaine solution (14 μg/mL) mixed with 0.01% acetic acid aqueous solution. Following the treatments, the locomotor activity of the juvenile zebrafish was evaluated over a 20 min observation period using the zebrafish tracking system from Viewpoint Life Sciences, Montreal, QC, Canada. Each larva was recorded ten times, with each recording lasting 2 min, and the total distances swum by the larva were ultimately documented. The movement of each larva was captured at 1 min intervals, accumulating a total of 20 recordings per individual [64].

### 4.11. Statistical Analysis

Statistical analyses were performed using GraphPad Prism (v8). Unless stated otherwise, data are expressed as the mean ± standard error of the mean (s.e.m.). For comparisons involving three or more groups, one-way analysis of variance (ANOVA) followed by Tukey’s multiple-comparison test was used.

## Data Availability

The original data presented in the study are included in the article; further inquiries can be directed to the corresponding author.

## Supplementary Materials

The following supporting information can be downloaded at: https://www.mdpi.com/article/10.3390/md23030102/s1, Figure S1: Fingerprint of crude venom of *C. tesselatus*; Figure S2: Fingerprint of crude venom of *C. generalis*; Figure S3: Fingerprint of crude venom of *C. caracteristicus*; Figure S4: Fingerprint of crude venom of *C. betulinus*; Figure S5: Fingerprint of crude venom of *C. quercinus*; Table S1: Similarity of crude venom fingerprints of 10 batches of *C*. *caracteristicus*; Table S2: Similarity of crude venom fingerprints of 10 batches of *C. betulinus*; Table S3: Similarity of crude venom fingerprints of 10 batches of *C. generalis*; Table S4: Similarity of crude venom fingerprints of 10 batches of *C. quercinus*; Table S5: Similarity of crude venom fingerprints of 10 batches of *C. tesselatus*.
