# Supplementary material for: Fingerprint Analysis and Comparison of Activity Differences of Crude Venom from Five Species of Vermivorous Cone Snail in the South China Sea
Source: Mar Drugs. 2025 Feb 25;23(3):102. doi: 10.3390/md23030102 (PMC11943727; doi:10.3390/md23030102)
Supplement: Supplementary file 1 [file marinedrugs-23-00102-s001.zip › Supplementary chart.pdf]

Supporting Information

For

Fingerprint Analysis and Comparison of Activity Differences of Crude Venom from Five Species of Cone Snail in the South China Sea

Shibo Sun, Yanling Liao, Jinxing Fu, Yanxia Liang, Yurong Chen, Kailin Mao*, Bingmiao Gao*

Engineering Research Center of Tropical Medicine Innovation and Transformation of Ministry of Education, Hainan Key Laboratory for Research and Development of Tropical Herbs, International Joint Research Center of Human-machine Intelligent Collaborative for Tumor Precision Diagnosis and Treatment of Hainan Province, School of Pharmacy, Hainan Medical University, Haikou 571199, China; sunshibo@hainmc.edu.cn (S.S.); liaoyanling@hainmc.edu.cn (Y.L.); hy0207145@muhn.edu.cn (J.F.);15708919030@163.com (Y.L.); hy0215005@muhn.edu.cn (Y.C.).

*Correspondence: maokailin@muhn.edu.cn(K.M.); gaobingmiao@hainmc.edu.cn (B.G.).

Supplement figures:

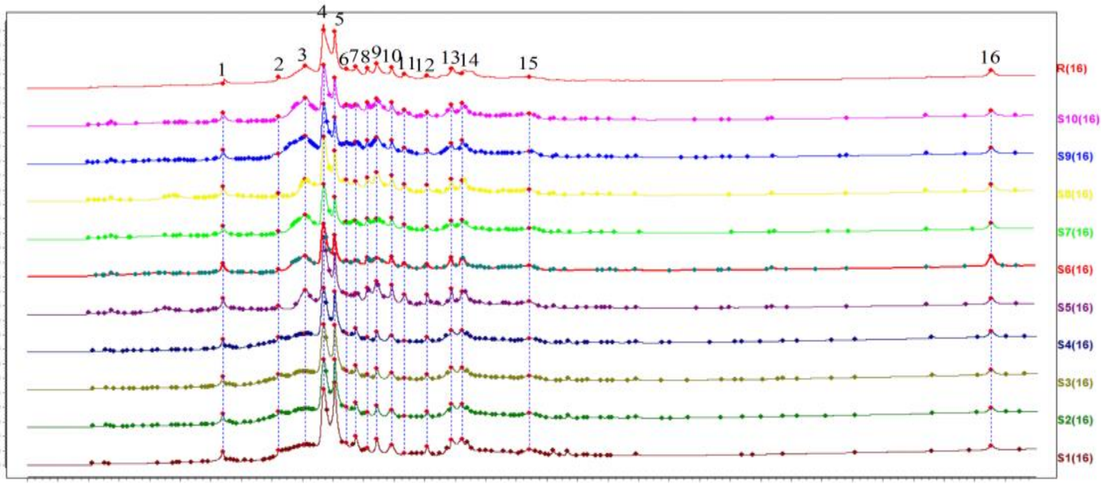

Figure S1. Fingerprint of crude venom of *C. tesselatus*. R is the reference map; S is the sample map. The numbers in brackets indicate the number of common peaks.

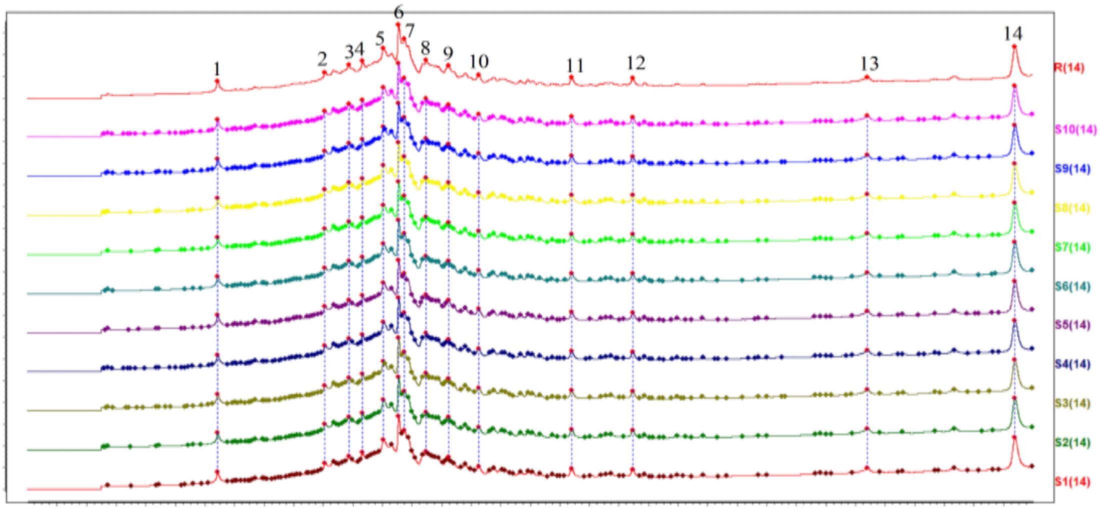

Figure S2. Fingerprint of crude venom of *C. generalis*. R is the reference map; S is the sample map. The numbers in brackets indicate the number of common peaks.

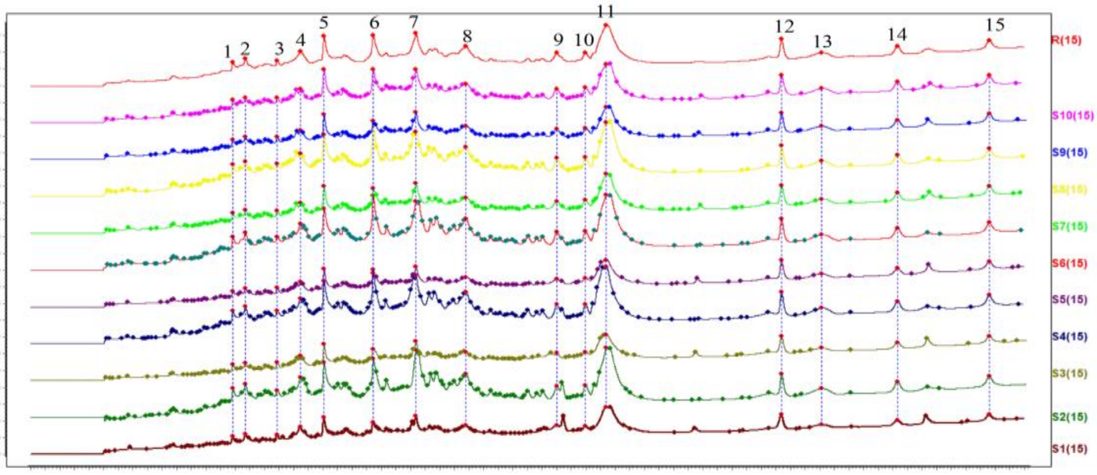

Figure S3. Fingerprint of crude venom of *C. caracteristicus*. R is the reference map; S is the sample map. The numbers in brackets indicate the number of common peaks.

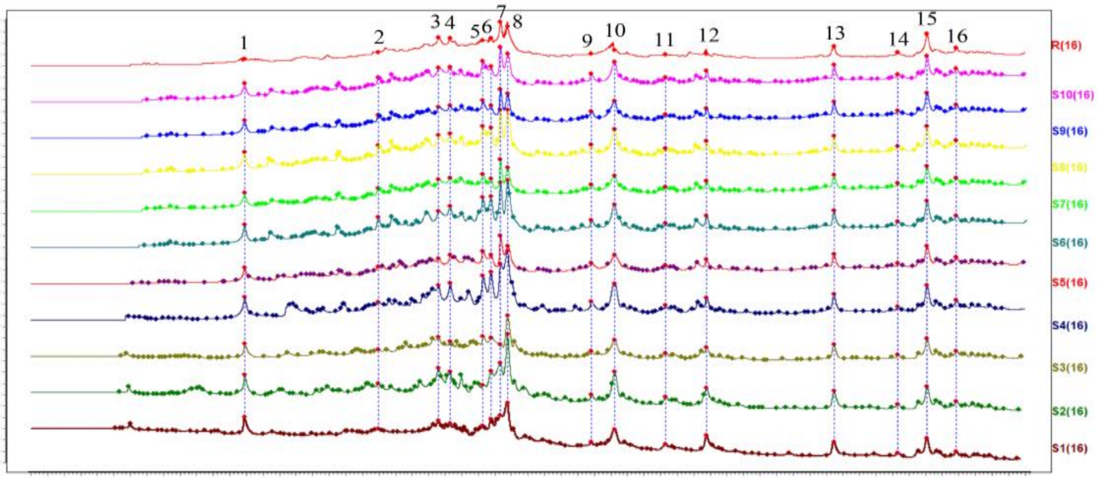

Figure S4. Fingerprint of crude venom of *C. betulinus*. R is the reference map; S is the sample map. The numbers in brackets indicate the number of common peaks.

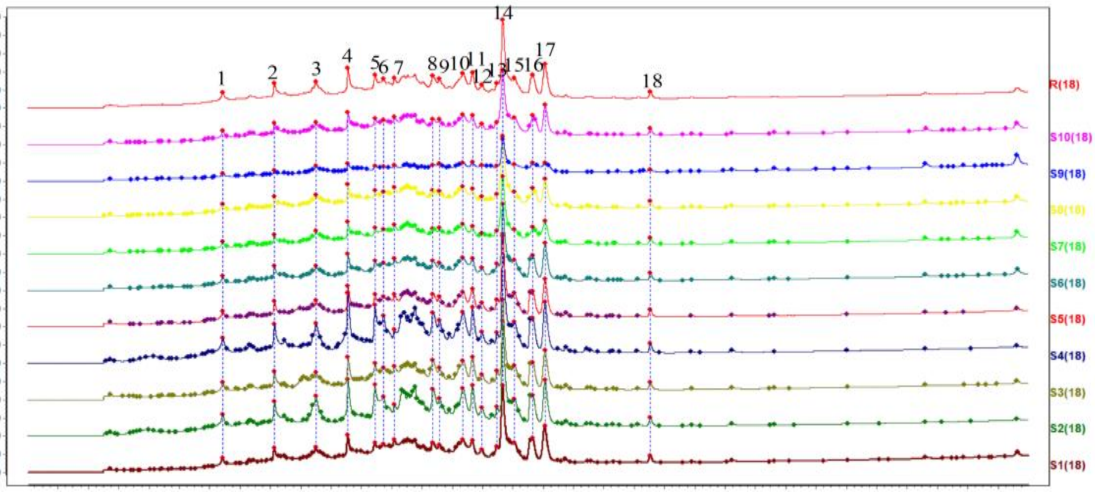

Figure S5. Fingerprint of crude venom of *C. quercinus*. R is the reference map; S is the sample map. The numbers in brackets indicate the number of common peaks.

Supplementary table:

Table S1. Similarity of crude venom fingerprints of 10 batches of *C. caracteristicus*.

	S1	S2	S3	S4	S5	S6	S7	S8	S9	S10	R
S1	1	0.920	0.933	0.893	0.872	0.901	0.891	0.895	0.890	0.890	0.935
S2	0.920	1	0.948	0.920	0.942	0.929	0.970	0.966	0.945	0.966	0.974
S3	0.933	0.9480	1	0.931	0.951	0.937	0.955	0.965	0.955	0.955	0.975
S4	0.893	0.9200	0.931	1	0.964	0.956	0.950	0.960	0.950	0.954	0.969
S5	0.872	0.9420	0.951	0.964	1	0.961	0.989	0.981	0.986	0.983	0.983
S6	0.901	0.9290	0.937	0.956	0.961	1	0.969	0.980	0.977	0.967	0.980
S7	0.891	0.9700	0.955	0.950	0.989	0.969	1	0.993	0.992	0.988	0.990
S8	0.895	0.9660	0.965	0.960	0.981	0.980	0.993	1	0.989	0.989	0.993
S9	0.890	0.9450	0.955	0.950	0.986	0.977	0.992	0.989	1	0.974	0.986
S10	0.890	0.9660	0.955	0.954	0.983	0.967	0.988	0.989	0.974	1	0.988
R	0.935	0.9740	0.975	0.969	0.983	0.980	0.990	0.993	0.986	0.988	1

Table S2. Similarity of crude venom fingerprints of 10 batches of *C. betulinus*.

	S1	S2	S3	S4	S5	S6	S7	S8	S9	S10	R
S1	1	0.980	0.984	0.981	0.992	0.998	0.983	0.985	0.955	0.987	0.997
S2	0.980	1	0.974	0.998	0.976	0.980	0.962	0.960	0.935	0.962	0.987
S3	0.984	0.974	1	0.973	0.996	0.986	0.973	0.973	0.954	0.967	0.990
S4	0.981	0.998	0.973	1	0.977	0.984	0.962	0.963	0.939	0.965	0.989
S5	0.992	0.976	0.996	0.977	1	0.992	0.982	0.982	0.958	0.976	0.995
S6	0.998	0.980	0.986	0.984	0.992	1	0.980	0.984	0.954	0.985	0.997
S7	0.983	0.962	0.973	0.962	0.982	0.980	1	0.997	0.935	0.972	0.986
S8	0.985	0.960	0.973	0.963	0.982	0.984	0.997	1	0.934	0.975	0.987
S9	0.955	0.935	0.954	0.939	0.958	0.954	0.935	0.934	1	0.968	0.962
S10	0.987	0.962	0.967	0.965	0.976	0.985	0.972	0.975	0.968	1	0.986
R	0.997	0.987	0.990	0.989	0.995	0.997	0.986	0.987	0.962	0.986	1

Table S3. Similarity of crude venom fingerprints of 10 batches of *C. generalis.*

	S1	S2	S3	S4	S5	S6	S7	S8	S9	S10	R
S1	1	0.990	0.991	0.993	0.925	0.933	0.920	0.950	0.905	0.842	0.975
S2	0.990	1	0.999	0.998	0.927	0.923	0.906	0.940	0.899	0.799	0.968
S3	0.991	0.999	1	0.999	0.926	0.922	0.907	0.942	0.898	0.802	0.968
S4	0.993	0.998	0.999	1	0.939	0.935	0.922	0.953	0.916	0.828	0.978
S5	0.925	0.927	0.926	0.939	1	0.977	0.981	0.959	0.979	0.921	0.982
S6	0.933	0.923	0.922	0.935	0.977	1	0.975	0.956	0.972	0.912	0.978
S7	0.920	0.906	0.907	0.922	0.981	0.975	1	0.962	0.976	0.939	0.977
S8	0.950	0.940	0.942	0.953	0.959	0.956	0.962	1	0.956	0.916	0.983
S9	0.905	0.899	0.898	0.916	0.979	0.972	0.976	0.956	1	0.926	0.971
S10	0.842	0.799	0.802	0.828	0.921	0.912	0.939	0.916	0.926	1	0.917
R	0.975	0.968	0.968	0.978	0.982	0.978	0.977	0.983	0.971	0.917	1

Table S4. Similarity of crude venom fingerprints of 10 batches of *C. quercinus.*

	S1	S2	S3	S4	S5	S6	S7	S8	S9	S10	R
S1	1	0.953	0.993	0.987	0.986	0.934	0.948	0.943	0.984	0.981	0.986
S2	0.953	1	0.950	0.965	0.940	0.987	0.974	0.996	0.959	0.957	0.988
S3	0.993	0.95	1	0.988	0.987	0.938	0.940	0.943	0.972	0.968	0.983
S4	0.987	0.965	0.988	1	0.987	0.961	0.955	0.959	0.969	0.970	0.991
S5	0.986	0.940	0.987	0.987	1	0.937	0.942	0.930	0.953	0.952	0.976
S6	0.934	0.987	0.938	0.961	0.937	1	0.958	0.989	0.927	0.926	0.976
S7	0.948	0.974	0.940	0.955	0.942	0.958	1	0.971	0.949	0.958	0.977
S8	0.943	0.996	0.943	0.959	0.930	0.989	0.971	1	0.952	0.952	0.983
S9	0.984	0.959	0.972	0.969	0.953	0.927	0.949	0.952	1	0.997	0.982
S10	0.981	0.957	0.968	0.970	0.952	0.926	0.958	0.952	0.997	1	0.982
R	0.986	0.988	0.983	0.991	0.976	0.976	0.977	0.983	0.982	0.982	1

Table S5. Similarity of crude venom fingerprints of 10 batches of *C. tesselatus.*

	S1	S2	S3	S4	S5	S6	S7	S8	S9	S10	R
S1	1	0.945	0.924	0.945	0.999	0.934	0.951	0.945	0.924	0.945	0.972
S2	0.945	1	0.99	0.997	0.946	0.948	0.945	0.932	0.99	0.997	0.994
S3	0.924	0.99	1	0.99	0.925	0.929	0.924	0.99	0.989	0.99	0.986
S4	0.945	0.997	0.99	1	0.946	0.946	0.945	0.997	0.99	0.987	0.994
S5	0.999	0.946	0.925	0.946	1	0.999	0.999	0.946	0.925	0.946	0.973
S6	0.95	0.948	0.929	0.946	0.999	1	0.978	0.948	0.929	0.946	0.974
S7	0.94	0.945	0.924	0.945	0.999	0.984	1	0.945	0.924	0.945	0.972
S8	0.945	0.95	0.99	0.997	0.946	0.948	0.945	1	0.99	0.997	0.994
S9	0.924	0.99	0.965	0.99	0.925	0.929	0.924	0.99	1	0.99	0.986
S10	0.945	0.997	0.99	0.943	0.946	0.946	0.945	0.997	0.99	1	0.994
R	0.972	0.994	0.986	0.994	0.973	0.974	0.972	0.994	0.986	0.994	1
